# ETV2/ER71, the key factor leading the paths to vascular regeneration and angiogenic reprogramming

**DOI:** 10.1186/s13287-023-03267-x

**Published:** 2023-03-16

**Authors:** Tae Min Kim, Ra Ham Lee, Min Seong Kim, Chloe A. Lewis, Changwon Park

**Affiliations:** 1 Graduate School of International Agricultural Technology and Institutes of Green-Bio Science and Technology, Seoul National University, 1447 Pyeongchang-daero, Pyeongchang, Gangwon-do 25354 Republic of Korea; 2 Department of Molecular and Cellular Physiology, Louisiana State University Health Science Center, 1501 Kings Highway, Shreveport, LA 71103 USA

**Keywords:** ER71/ETV2, Endothelial cells, Vascular regeneration, Direct cell reprogramming

## Abstract

Extensive efforts have been made to achieve vascular regeneration accompanying tissue repair for treating vascular dysfunction-associated diseases. Recent advancements in stem cell biology and cell reprogramming have opened unforeseen opportunities to promote angiogenesis in vivo and generate autologous endothelial cells (ECs) for clinical use. We have, for the first time, identified a unique endothelial-specific transcription factor, ETV2/ER71, and revealed its essential role in regulating endothelial cell generation and function, along with vascular regeneration and tissue repair. Furthermore, we and other groups have demonstrated its ability to directly reprogram terminally differentiated non-ECs into functional ECs, proposing ETV2/ER71 as an effective therapeutic target for vascular diseases. In this review, we discuss the up-to-date status of studies on ETV2/ER71, spanning from its molecular mechanism to vasculo-angiogenic role and direct cell reprogramming toward ECs. Furthermore, we discuss future directions to deploy the clinical potential of ETV2/ER71 as a novel and potent target for vascular disorders such as cardiovascular disease, neurovascular impairment and cancer.

## Introduction

Until recent decades, the long-standing view of development, whereby cells lose their differentiation potential throughout development and developed cells have very limited capabilities to rejuvenate or become other cell types, had discouraged attempts to generate pluripotent/multipotent stem cells from somatic cells for the purpose of replacing damaged tissues or failing organs, an ultimate goal of biomedical research [1]. Additionally, the idea that replenishing defective tissues or organs in vivo through transdifferentiation from other cell types with a relatively large abundance such as fibroblasts could be a testable option for replacement therapy had been largely denied. However, recent studies have revealed the possibility of changing already determined cellular identities to those of other cell types, in a targeted manner. Overexpression of transcription factors can induce pluripotency in somatic cells (i.e., induced pluripotent stem cells, iPSCs) [2] or directly change one cell type into another (direct reprogramming or direct conversion) [3, 4]. The latter has several advantages over iPSCs in that it is simple, fast and has very low risk of tumorigenesis [4].

Transcription factors are proteins regulating a wide array of target genes to govern certain developmental pathways (e.g., endothelial lineage specification) or biological events (e.g., inflammation). In particular, pioneer transcription factors such as OCT4, FOXA, ASCL1 and PU.1 can open closed chromatin and induce the expression of target genes in ectopic cells, forcing them to acquire another identity [5]. In endothelial cells (ECs), ETV2 (also known as ER71), a member of the E26 transformation-specific or E26-specific sequence (ETS) transcription factor family, is considered a pioneer factor [6]. Upon overexpression, ETV2 can initiate the de novo generation of ECs from mouse embryonic stem cells (mESCs) [7, 8], and ETV2 alone is sufficient to directly convert fully differentiated non-ECs to functional ECs [9, 10]. Despite its promising outcomes, the translation of the therapeutic potential of ETV2 into clinical use for vascular diseases faces several key hurdles. There is a lack of a useful mode of delivery of ETV2 for clinical use with non-viral, non-genetic, non-inflammatory methods, as well as efficient and specific in vivo delivery tools, and the heterogeneous nature of reprogrammed ECs hinders its application. In the first part of this review, we describe the functions of ETV2 in vascular system establishment in developing embryos. Next, we discuss direct cell reprogramming mediated by ETV2 and its potential therapeutic functions in vivo. In the last section, we focus on future directions for the use of ETV2 as a therapeutic vehicle considering its clinical applicability.

## Critical role of ETV2 in establishing the cardiovascular system in developing embryos

The ETS transcription factors are known to play critical roles during EC specification and the establishment of the cardiovascular system [11]. We and other laboratories have identified *Etv2* (also known as *Er71* or *ets1-related protein*, *etsrp*) as a novel ETS member with an irreplaceable role in embryonic vessel formation [7, 8, 12–14]. The significance of *Etv2* in vessel development was first reported in a zebrafish study using a *cloche* mutant, with no manifestations of blood, endothelial and endocardial cell formation [12]. Microarray analyses between normal zebrafish and *cloche* mutants revealed several differentially regulated endothelial-specific genes, among which *etsrp* was identified as a novel endothelial gene. The knockdown of *etsrp* via morpholino (MO) injection resulted in a complete loss of blood vessel formation and marked reduction in mRNA expression of endothelial markers. In contrast, the forced expression of *etsrp* mRNA in *cloche* mutants led to a restoration of critical endothelial genes (*flk1, scl, fli1* and *cdh5*), indicating that *etsrp* is essential for establishing embryonic vasculature [13]. The critical function of *Etv2* in early endothelial lineage was corroborated in mammals as demonstrated by *Etv2*-knockout mice and mouse embryonic stem cell (mESC) differentiation approaches [7, 8, 14, 15]. Similar to the findings observed with *etsrp* MO in zebrafish, *Etv2*-deficient mouse embryos have no detectable hematopoietic or ECs, resulting in early embryonic lethality between embryonic day (E) 9.5 and E10.5. Furthermore, *Etv2* overexpression in differentiating mESCs led to an increase in fetal liver kinase-1 (FLK1, also known as VEGFR2)-expressing cells, early mesenchymal/endothelial progenitor cells that can differentiate into hematopoietic and ECs [16]. Interestingly, *Etv2* overexpression induced the de novo generation of FLK1^+^ cells in serum-free differentiation conditions, where mESCs differentiate only up to the embryonic germ layer stage. Genome-wide transcriptome, proteomics analyses and chromatin immunoprecipitation sequencing have demonstrated that ETV2 alone or often together with other transcription factors such as OVOL2 [17], GATA2 [18] and TET2 [19] directly binds its numerous endothelial target genes including *Flk1, Cdh5, Tek, Flt4* and *Egfl7* [20, 21] and activates their expression, driving EC generation in developing embryos.

Other than its role in endothelial fate acquisition, a recent study suggests that ETV2 may play a critical role in endothelial progenitor migration for embryonic vessel formation [22]. ETV2 was found to directly binds the ETS consensus sequences within the promotor of *RhoJ*, a Rho-related GTP-binding protein. *Etv2* overexpression led to a robust *RhoJ* expression, but its message was abolished in embryoid bodies (differentiated cell aggregates of ESCs) lacking *Etv2*. In mouse embryonic fibroblasts (MEFs), ETV2-mediated migration was inhibited by *RhoJ* knockdown, which was partially rescued by the introduction of *Etv2* into MEFs [22]. Regarding the protein family of Rho GTPases, another study reported it as a potential direct target of ETV2 in FLK1^+^PDGFRα^−^ endothelial/hematopoietic progenitors in mESCs [20]. Additionally, *RhoJ* was shown to be directly activated downstream of ERG [23], which is also an ETS transcription factor acting downstream of ETV2 [20]. These observations support the pro-angiogenic role of ETV2 through *RhoJ* in regulating cell migration/motility. Similarly, we have shown that ETV2 can regulate ECs motility through *Myct1*, a novel endothelial gene directly activated by ETV2 (see below for further information) [24]. Altogether, studies over the past decade have clearly indicated a potent and indispensable function of ETV2 in ECs. For more details on the role of ETV2 in early development and its underlying molecular mechanisms, readers are referred to our previous review [25, 26].

## ETV2 is an emerging target for direct reprogramming of non-endothelial cells into endothelial cells

Studies thus far have posited ETV2 as a master regulator of EC development. One important feature of ETV2 is that its expression becomes undetectable beyond E11.5 during mouse embryogenesis [7, 8, 14, 15]. In postnatal life, *Etv2* expression is markedly observed in the testis [27] and remains silent in most tissues including quiescent ECs in adults [28], suggesting that the sustained expression of *Etv2* might have detrimental biological consequences in a steady state. Indeed, when ECs were forced to express *Etv2* throughout development*,* vessels of the mice (*Tie2Cre;RosaR26-Etv2*) were dilated, resulting in lethality [29]. Furthermore, ectopic *Etv2* expression induced endothelialization in non-endothelial compartments [29], accounting for its transient expression. However, this seemingly undesirable ETV2 function could be beneficial when generating ECs through the directed differentiation of stem cells or reprogramming cells for cell replacement therapy. In the following sections, we discuss the translational applicability of ETV2 with an emphasis on EC generation from diverse cell sources for vascular-related disease.

John Gurdon’s historical discoveries that unfertilized enucleated frog eggs transplanted with the nucleus of a mature intestinal epithelial cell can generate a frog [30] challenged the classical view that cells lose their potential upon differentiation due to irreversible genomic modifications, which is well represented by Waddington’s famous rolling ball theory [31]. These provocative findings had gained support from the generation of Dolly the sheep, whereby a mammary gland cell’s nucleus was transferred into an enucleated egg [32]. Taken together with the reports that somatic cells such as fibroblasts and thymocytes fused with human ESCs (hESCs) generate hybrid cells with an ES character [33, 34], these studies suggest not only that the genome of a fully mature cell has totipotency or pluripotency, but also that a fully differentiated cell can be converted into an embryonic stage through appropriate environments or stimuli. Indeed, Yamanaka and colleagues have shown that somatic cells (mouse embryonic and adult tail-tip fibroblasts) can be reprogrammed into ES-like cells, known as iPSCs, through several key transcription factor overexpression [35, 36]. Twenty-four initially selected genes were tested for their ability to induce pluripotency in somatic cells as demonstrated by cell morphology, gene expression, differentiation potential and DNA methylation profile (bisulfite genomic sequencing). They were finally narrowed down to four factors; *Oct3/4, Sox2, c-Myc* and *Klf4*. Upon transplantation into athymic nude mice, the cells reprogrammed by the four factors (also known as Yamanaka factors) developed teratomas containing derivatives of three embryonic germ layers, confirming the bona fide pluripotency of reprogrammed cells. Consistent results were obtained with human somatic cells and the similar combinations of these transcription factors [36–38], opening a new era of cell reprogramming. These findings are particularly important for clinical use as iPSCs can ideally avoid ethical issues and the immune compatibility problems of hESCs.

In parallel, sporadic observations that certain types of somatic cells can acquire another identity have been reported. Overexpression of the eyeless transcription factor, *Paired Box 6* (*Pax6*), can lead to *Drosophila* ectopic eye development on sites including the wings, legs and antennae [39]. *Myogenic Differentiation 1* (*MyoD*), a member of the basic helix–loop–helix family of transcription factors, plays important functions in muscle differentiation [40]. Interestingly, non-myogenic cells, such as fibroblasts, and melanoma and neuroblastoma cells transfected with *MyoD* were converted into cells expressing muscle-specific genes and displaying muscular cell morphology [41]. Other studies have also shown the conversion of cell identity within the same cell lineages such as that of B cells into macrophages by *CCAAT Enhancer Binding Protein alpha* (*C/EBPα*) and CCAAT Enhancer Binding Protein beta (*C/EBPβ*) [42], and that of myeloblasts into eosinophils, thromboblasts and erythroblasts by *GATA1* (GATA Binding Protein 1) [43]. Similarly, conversion of pancreatic exocrine cells into insulin-secreting β-like cells occurred in vivo when transcription factors *Ngn3, Pdx1* and *Mafa* were introduced into mice [44]. These results strongly suggest that fully differentiated somatic cells can be converted or reprogrammed into other cell types through the overexpression of lineage-specific transcription factors or master regulators of specific cell lineages.

Encouraged by studies on iPSCs and cell type conversion between somatic cells, systematic approaches have attempted to directly convert diverse somatic cell types into other cell types through key transcriptional regulators. This strategy enables bypassing the pluripotent stage, thereby substantially reducing the tumorigenic potential and shortening procedural times, which are the major advantages of the direct cell conversion or reprogramming method over iPSC technology [4]. For example, a combination of the nervous system-specific transcription factors (*Ascl1, Brn2* and *Myt1l*) directly converted fibroblasts into neurons. *Ascl1* alone was sufficient to induce immature neural function, but its combined expression with *Brn2* and *Myt1l* generated mature neuronal cells with higher efficiency [45]. Induced hepatic cells can be directly generated from mouse fibroblasts when hepatic lineage-specific transcription factors (transduction of *Gata4, Hnf1α, Foxa3* and *p19* Arf inactivation) are overexpressed [46]. Another study has shown that the combination of *Gata4*, *Mef2c* and *Tbx5* can directly reprogram postnatal mouse fibroblasts and tail-tip fibroblasts into functional cardiomyocytes [47]. When fibroblasts transduced with these factors were transplanted into a mouse heart, they differentiated into cardiomyocyte-like cells. In a similar study, GAMT transcription factors (*Gata4, Hand2, Mef2c* and *Tbx5*) were used to directly convert adult mouse tail-tip and cardiac fibroblasts into cells with a cardiac phenotype [48]. Furthermore, the authors showed that the delivery of the four factors into the mouse heart enables non-cardiomyocytes to become cardiomyocyte-like cells and improves cardiac functions after myocardial infarction (MI). Please refer other reviews for further reading on iPSCs [2] and direct cell reprogramming to non-ECs [3, 4].

Accordingly, extensive efforts have been made to directly generate functional ECs from non-ECs. The first successful attempt was reported by Rafii and colleagues [49]. Lineage-committed human amniotic fluid-derived cells (ACs), defined as EpCAM^+^TRA1-81^−^c-KIT^−^ and EpCAM^−^TRA1-81^−^c-KIT^−^, were directly reprogrammed into ECs through a first wave of transient *Etv2* overexpression, followed by constitutive *Erg* and *Fli1* expression, and TGF-β pathway inhibition [49]. Additionally, the authors showed that the induced vascular ECs (iVECs) exhibit mature EC characteristics and form stable and functionally perfused vessels in vivo, as shown by matrigel plug assay and hepatectomy-induced regrowth of sinusoidal vessels. Shortly after, direct reprogramming of mouse adult dermal fibroblasts to ECs was achieved by overexpressing five transcription factors: *Foxo1, Klf2, Tal1, Lmo2* and *Etv2*. Importantly, the reprogrammed ECs improved perfusion recovery when cells were transplanted into a mouse model of hindlimb ischemia [50]. Although promising, these approaches have limited therapeutic applicability. Access to the ACs is invasive, and iVECs are incompatible with autologous cell transplantation. Additionally, the reprogramming requires multiple transcription factors in the form of viral particles, which could complicate long-term outcome and elevate genetic burdens, leading to genomic instability. Given that the transcription factors used for direct reprogramming are ETV2 targets, two groups, including our team, have successfully demonstrated that the single factor, ETV2, can directly convert human dermal fibroblasts (HDFs) into cells with endothelial functionality. Morita et al. showed that transient *ETV2* expression in HDFs is sufficient to induce stable endothelial gene expression [9]. Additionally, the reprogrammed ECs were incorporated into host blood vessels in the Matrigel plug assay and enhanced blood flow recovery in mice undergoing ischemic insult. Independently, we also directly converted HDFs into functional ECs using *ETV2* only [10]. Interestingly, we found two successive stages of direct reprogramming: early and late-reprogrammed ECs. At the early stage, the reprogrammed ECs display immature EC characteristics but have vascular regeneration and tissue repair functions, as demonstrated through in vitro assays and an in vivo hindlimb ischemia model. The late stage reprogrammed ECs are generated when the early reprogrammed ECs are further cultivated with a boost of ETV2 expression together with valproic acid (VPA, a histone deacetylase inhibitor) treatment. Late-reprogrammed ECs has similar transcriptomes to those of mature ECs and produced nitric oxide and highly expressed CD31, which are representative mature EC markers. Importantly, *ETV2* expression in late-reprogrammed ECs was very low, meeting the criteria of mature ECs [28].

The potent direct cell reprogramming ability of ETV2 is also evident in other somatic cell types. The short-term expression of *ETV2* while inhibiting TGF-β signaling changed human adipose-derived stem cells (hADSC) into EC-like cells with durable vascular identity [51]. In this study, KDR/VEGFR2^+^ cells isolated from *ETV2*-transduced hADSCs in the presence of TGF-β inhibitor were cultured without the continued expression of *ETV2*. Transcriptome analyses revealed a shared transcriptome profile between the further cultured cells and mature ECs. The authors showed the therapeutic potential of the reprogrammed cells in promoting vascular repair. The same reprogramming scheme induced endothelial identity in human umbilical cord-derived mesenchymal stem cells [51]. Mouse adventitial SCA1^+^ progenitors transduced by ETV2 acquired endothelial characteristics of gene expression profile, phenotype and function [52]. When the reprogrammed SCA1^+^ cells were transplanted into wire-injured femoral arteries, the mice receiving the cells showed improved vascular remodeling due to reduced smooth muscle cell proliferation and enhanced reendothelialization [52]. Additionally, fast skeletal muscle cells in zebrafish injected with *Etv2* became ECs and then were incorporated into the vascular network. However, such a change was possible only when *Etv2* was injected into embryos in a limited developmental window (between 22 and 30 postfertilization) [53]. The C2C12 mouse myoblast cell line can also express endothelial genes in response to *Etv2*. Consistently, *Etv2*-deficient hematopoietic and vascular progenitors differentiated into skeletal muscle cells in zebrafish embryos [54]. Altogether, these results strongly suggest that ETV2 alone and often together with other signaling molecules can directly convert non-ECs into functional ECs, which could have therapeutic potential for treating cardiovascular disease. A summary of ETV2-mediated direct reprogramming to ECs is shown in Table 1.

**Table 1:** Summary of ETV2-mediated direct reprogramming to endothelial cells

Cell source	Delivery method	Factors	In vivo functional assay	Outcome	References
Human amniotic cells	Lentivirus	ETV2, FLI1, ERG1 and inhibition of TGFβ	Angiogenesis	Improved perfusion vessels	Ref. [49]
Mouse adult skin fibroblasts	Lentivirus	Foxo1, Etv2, Klf2, Tal1 and Lmo2	Hindlimb ischemia	Improved blood flow recovery	Ref. [50]
Human adult skin fibroblasts	Lentivirus	ETV2	Hindlimb ischemia	Improved perfusion vessels	Ref. [9]
Human dermal fibroblasts	Lentivirus	ETV2	Hindlimb ischemia	Direct vascular incorporation and angiogenesis	Ref. [10]
Human adipose-derived stem cells	Lentivirus	ETV2 and inhibition of TGFβ	Hindlimb ischemia	Promoted revascularization	Ref. [51]
Vascular adventitial Sca1 + progenitor cells	Adenovirus	ETV2	Femoral artery wire injury model	Improved vascular remodeling	Ref. [52]
Zebrafish fast muscle cells	Transgenic and Heat Shock	ETV2	Observational study (time lapse imaging/microangiography)	Integration of ECs into vascular network and subsequent blood circulation	Ref. [53]
Human dermal fibroblasts	Lentivirus	ETV2 and hypoxia conditions (5% oxygen)	Not conducted	Improved reprogramming efficacy vs normoxia	Ref. [55]

Studies have also reported that the use of ETV2 and hypoxia increases the reprogramming efficiency [55]. Fibroblasts transduced with *ETV2* under hypoxic conditions formed CD31^+^VEGFR2^+^ cells, and the reprogramming efficacy to endothelial progenitor cell was increased, compared with those under normoxic conditions. Intriguingly, Mathison et al. found that *ETV2*-overexpressing rat cardiac fibroblasts show increased cardiomyocyte markers *cTnT* and *Actc1* and higher expression of cardiac markers when additionally treated with *GMT* (*Gata4, Mef2c* and *Tbx5*) [56]. Human cardiac fibroblasts also expressed higher *cTnT* level when treated with *ETV2* and *GMT,* compared with cells treated with *GMT* alone. This study suggests that the transendothelial state induced by ETV2 overexpression induces cell reprogramming with high efficiency and represents a desirable cellular target for cardiac differentiation. Thus, it is tempting to speculate that the generation of EC subtypes such as arterial, venous and lymphatic ECs from fibroblasts can be efficiently achieved by ETV2 transduction, followed by that of EC subtype-specific transcription factors such as COUP-TFII for venous ECs and Prox1 for lymphatic ECs [16].

## Therapeutic potential of etv2 in pathophysiological angiogenesis

In postnatal tissues, angiogenesis occurs during the repair phase of injury after inflammation via EC proliferation, vessel sprouting and remodeling. Although quiescent in adults, ECs have the ability to rapidly proliferate upon various environmental cues such as hypoxia, inflammation or reactive oxygen species, all of which can be found in developmental angiogenesis [57]. The transient expression of *Etv2* only in early embryogenesis puzzles its potential role in postnatal angiogenesis despite its potent and specific function in EC generation and functionality. We provided the first evidence on *Etv2* function in adults, using a mouse model of hindlimb ischemia. *Etv2* expression was reactivated in hindlimb ECs upon ischemic injury [58]. Importantly, mice deficient in endothelial *Etv2* showed significantly compromised vessel formation in a series of acute injury models, including hindlimb ischemic injury, laser-induced eye injury and skin wounding. In contrast, the forced expression of *Etv2* in the mouse hindlimb under ischemia insults promoted blood perfusion recovery and new vessel generation. Considered along with findings of the significantly reduced necrosis and fibrosis in *Etv2* injected hindlimb muscles, these results indicate that *Etv2* plays essential roles in new vessel generation and tissue repair in response to ischemic damages in postnatal life. The potential therapeutic function of *Etv2* was also demonstrated in a murine model of MI. The delivery of lentiviral *Etv2* led to a significant improvement of cardiac functions and substantial induction of vessel formation in hearts with MI [59]. Interestingly, adeno-associated virus (AAV)-mediated *Etv2* delivery also reduced infarct size and enhanced cardiac functions, further supporting its therapeutic feasibility for clinical applications. It is noteworthy that the therapeutic role of *Etv2* in adult vessel repair shown in these two independent rodent models of hindlimb ischemia and MI may not be solely due to *Etv2*’s direct pro-angiogenic function in ECs, since anti-inflammatory and antifibrotic effects in injured tissue were also observed. Further in-depth studies on *Etv2* function in endothelial, parenchymal and other interstitial cells are warranted.

The tumor angiogenesis studies reinforce the therapeutic applicability of ETV2 in vascular-related disease. Conditional inhibition of *etsrp* in zebrafish receiving mouse B16 melanoma cells resulted in a marked decrease in tumor vessel growth, compared with that of control zebrafish [60]. Other investigations of various human malignant tumor tissues (lung, breast, prostate and colon) revealed that *ETV2* is expressed in tumor-associated ECs (TAECs), but not in ECs from healthy controls [61]. Consistently, *Etv2* expression was also evident in the TAECs of mice transplanted with Lewis lung carcinoma cells, while remaining silent in control mice ECs [61]. In agreement with these findings, the essential function of *Etv2* during tumor formation was confirmed in endothelial *Etv2*-deficient and *Etv2* siRNA-treated mice, as shown by a reduced tumor volume with a significant decrease in tumor vessel formation. The role of *Etv2* in pathological angiogenesis was also demonstrated in glioblastoma multiforme (GBM), a malignant tumor with high recurrence. Zhao et al. showed that *ETV2* expression positively correlated with GBM severity and that ETV2-positive tumor cells in high-grade GBM tissues expressed the endothelial marker CD31 [62]. Remarkably, ECs that coexpressed ETV2 and CD31 were more enriched in the core region of GBM tissue than in the superficial region, suggesting that TAECs in hypoxic locations have higher *ETV2* expression. *ETV2* could also transdifferentiate CD133^+^/Nestin^+^ GBM neural stem cells (NSCs) to EC-like cells, possibly by suppressing critical neural differentiation genes [62], which is consonant with ETV2’s role in converting non-ECs to cells with an endothelial functionality. These findings prompt the speculation that ETV2 could be used to turn devastating cancer or cancer stem cells into non-cancerous cells. A summary on the function of ETV2 in vivo is provided in Table 2.

**Table 2:** Experimental validation of ETV2 function in therapeutic angiogenesis

Animal model	Animal species	Delivery methods	Administration route and dose	Observation time points	Outcome	References
Hindlimb ischemia	Athymic nude mice	Lentivirus	Single IM injection of 25 µl (IFU 3 × 10^7^/ml) into the adductor muscle (4 sites, a total 100 µl per animal)	Day 7, 21, 28	Improved blood perfusion	Ref. [58]
Myocardial infarct	C57/BL6	Lentivirus	Single intra-myocardial injection of 5.6 × 10^6^ IFU per site, a total two sites	8 weeks after MI	Improved cardiac function Increased capillary density	Ref. [59]
Myocardial infarct	Fisher 344 rats	Adeno-associated virus	Single intra-myocardial injection of 1 × 10^12^ GC per site, two sites	8 weeks after MI	Improved cardiac function Less fibrosis	Ref. [59]
Stroke	C57/BL6	Nanochannel electroporation of plasmid DNA encoding EFF (Etv2, Foxc2, Fli1) into MEFs	Single intracranial (subarachnoid space) injection of EFF-transfected MEFs at day 7 of MCAO	Day 21 after MCAO	Enhanced cerebral vascularity, infarct resolution and motor activity	Ref. [112]
Syngenic tumor model	C57/BL6	ETV2 siRNA nanoparticle	Repeated IV injection of 1 nmol per mouse at day 9–17 after SC transplantation of LLC, a total 5 times, EOD	Day 19 after LLC transplantation	Reduction in tumor size, less vessels	Ref. [61]
Heterotopic human GBM tumor	NOD/SCID mice	Lentiviral delivery of ETV2-specific gRNA/Cas9 into GBM cells	SC transplantation of ETV2-disrupted GBM cells (5 × 10^6^ per mouse)	2 months after GBM transplantation	Reduction in tumor size and hCD31^+^/KI67^+^ TDECs	Ref. [62]

*EFF* Etv2/Foxc2/Fli1; *EOD* Every other day; *GBM* Glioblastoma; *GC* Genome copy; *gRNA* Guide RNA; *IFU* Infectious unit; *IM* Intramuscular; *IV* Intravascular; *LLC* Lewis lung carcinoma; *MCAO* Middle cerebral artery occlusion; *MEFs* Mouse embryonic fibroblasts; *MI* Myocardial infarct; *NOD/SCID* Non-obese diabetic/severe combined immune deficiency; *SC* Subcutaneous

To understand the detailed molecular mechanism by which ETV2 controls EC generation and function, many groups have attempted to identify the direct downstream targets of ETV2, revealing several key factors such as *Flk1, Cdh5, Tie2, Tal1, Lmo2* and *RhoJ* [20, 22]. Recently, our team has identified a novel and endothelial-specific gene, *Myct1 *(*Myc target* 1), as a direct bona fide effector downstream of ETV2. Based on the results of gene expression profiling from various solid tumor tissues of patients, and subsequent comparisons with the target genes of ETV2, we hypothesized that *Myct1* could be directly activated by ETV2 [24]. Single-cell sequencing analyses confirmed its specific expression in ECs, and subsequent assays showed that ETV2 can directly bind to the promoter region of *Myct1*, activating its expression. We further found that *Myct1* is expressed in both plasma membrane and the Golgi complex and regulates motility and tight junctional integrity of ECs. Importantly, *Myct1* inhibition (i.e., *Myct1* global knockout and *Myct1* endothelial knockout) led to a reduction in tumor growth and angiogenesis, reminiscent of *Etv2*-deficient tumor vessel [61]. Interestingly, the lack of *Myct1* in TAECs augmented antitumor immunity, as shown by the enhanced transendothelial migration of cytotoxic T lymphocytes and M1 macrophage polarization in the absence of endothelial *Myct1*. The combined inhibition of *Myct1*, programmed death 1 (PD1) and VEGFR2 signaling completely blocked tumor growth, whereas cotreatment with anti-PD1 and anti-VEGFR2 blocking antibodies failed to induce tumor regression. Collectively, these findings suggest that the ETV2-MYCT1 axis plays essential roles in arborizing tumor vessels and regulating tumor immunity, indicating that in-depth analysis of ETV2 functions could open unforeseen opportunities in the treatment of vascular disease such as cancer, MI and critical limb loss.

## ETV2 for directed pluripotent stem cell differentiation into hematoendothelial cells

**Fig. 1:**
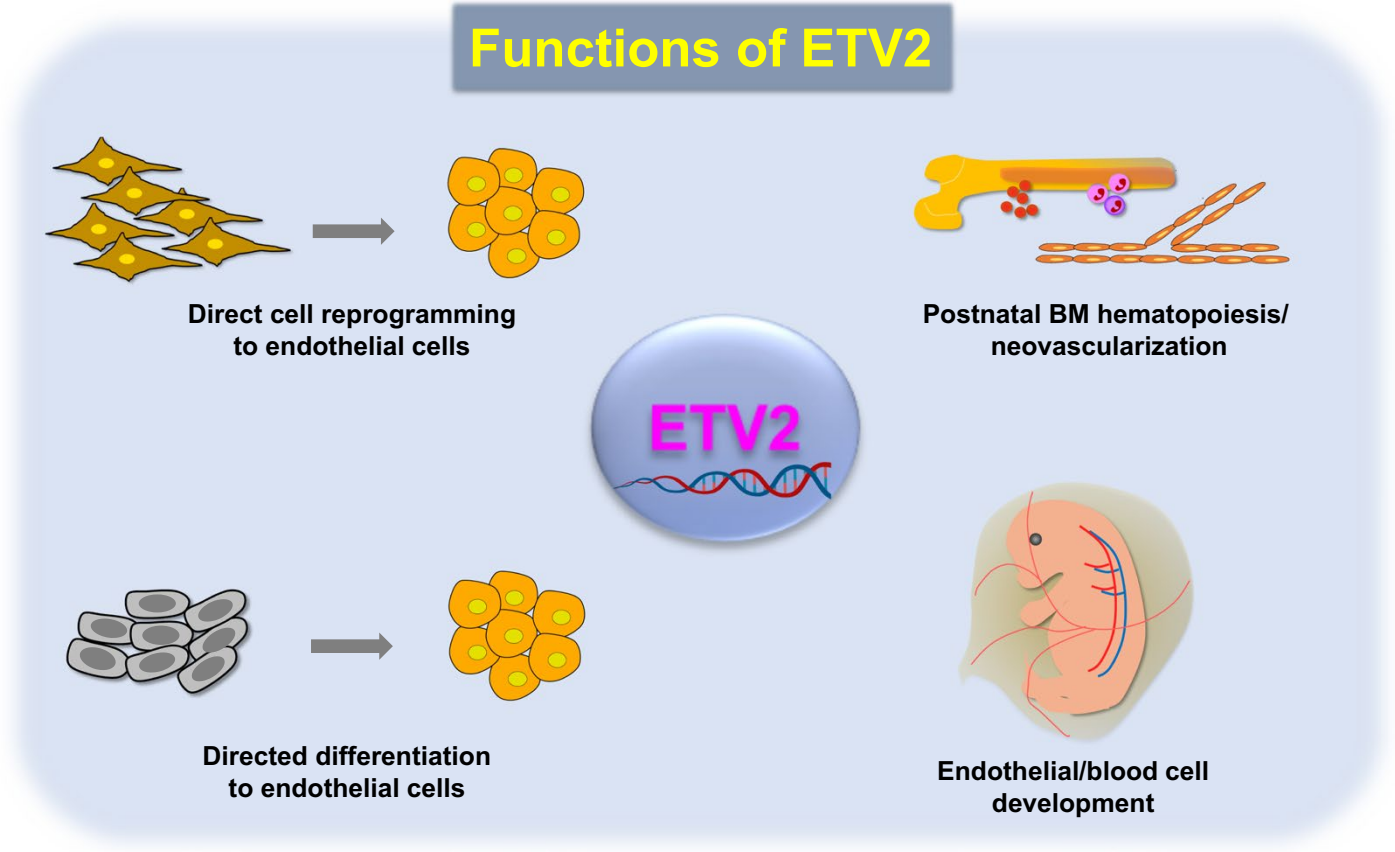
Schematic diagram of ETV2 function. ETV2 has multiple functions in the regulation of the cardiovascular system. The development of hematopoietic and endothelial cells is critically dependent on ETV2 as evidenced by a complete lack of both lineages in *Etv2* deficient embryos. In adults, endothelial ETV2 is required for new vessel formation in response to injury. Furthermore, ECs lacking *Etv2* have impaired ability to support tumor growth. In bone marrow (BM), ETV2 regulates hematopoietic stem cell proliferation, contributing to BM hematopoiesis. Recent studies have shown that ETV2 can directly generate ECs from non-ECs and PSCs. Created in BioRender.com

Pluripotent stem cells (iPSCs and ESCs) are undifferentiated pluripotent cells that can provide scalable amount of various types of differentiated target cells for therapeutic and experimental purposes [63]. It has been shown that hematopoietic differentiation of PSCs by *Etv2/Gata2* overexpression was preceded by transient endothelial phenotypes (CDH5^+^/CD73^−^/CD43^−^ cells), and they became hematopoietic cells (CD43^+^ cells) within 2 days [64]. The transfection of *Etv2/Gata2* modified mRNA (mmRNA) activated the hematoendothelial differentiation in hESCs, resulting in a similar number of colony-forming cells to that obtained by lentiviral *ETV2/GATA2* [64]. A recent study also demonstrated that untranslated region (UTR)-*ETV2* mmRNA alone was capable of inducing PSCs into CDH5^+^ cells within 1–2 days, after which they became myeloid progenitors and, subsequently, functional neutrophils [65]. Therefore, these two studies suggest that ETV2-directed hematoendothelial cells can be obtained from PSCs without pan-mesodermal stages, providing an efficient method for generating hematoendothelial lineages. More recently, Wang et al. have reported a two-step method for generating ECs from iPSCs; iPSCs were first converted into mesodermal progenitor cells (MPCs) by Wnt/Nodal signaling activation using a glycogen synthase kinase 3 (GSK3) inhibitor (CHIR99021). The resulting MPCs were then transfected with *ETV2* mmRNA [66]. This strategy allowed the generation of CDH5^+^/CD31^+^ induced ECs (iECs) (approximately 95% positive) after 48 h of *ETV2* mmRNA delivery. These iECs robustly expanded for 3 weeks yielding approximately 70-fold increase. In contrast, bypassing the MPC stage by transfection of *ETV2* mmRNA into iPSCs (not into MPCs) resulted in a marked decrease in iEC proliferation, yielding only twofold increase in 2-week period. In vivo, the number of functional, perfusable vessels surrounded by α-smooth muscle actin^+^ perivascular layer was higher in mice injected with iECs produced from a two-step protocol compared with that of mice receiving those from a one-step protocol. The results of this study underpin the importance of timely ETV2 expression in hematoendothelial cell progenitors for proper EC development. Furthermore, these results suggest the feasibility of *ETV2* mmRNA for target cell generation (directed differentiation, direct cell reprogramming in vitro and in vivo) for clinical application due to its minimal mutagenic potential, unlike that of viral gene delivery. A summary on the function of ETV2 is provided in Fig. 1.

## Future perspectives on etv2 as a therapeutic vehicle for vascular disease

### Advantages of ETV2-mediated vessel recovery

Currently, medications for vessel diseases include (para)sympathomimetics or chemicals for vasodilation, blood pressure reduction, cholesterol level reduction, clot prevention, as well as other drugs used for treating diabetes and hypertension [67]. As discussed, ETV2 is an indispensable transcription factor that can directly up-regulate wide arrays of genes necessary for early vessel development, reprogramming and neovascularization in adult vessels under pathophysiological conditions. Thus, it can be expected that ETV2 alone can reactivate the expression of multiple essential genes which are required for vessel regrowth unlike certain angiogenic factors such as VEGF that can only activate limited sets of genes. Also, ETV2 promotes the mesodermal progenitor cells to express FLK1 (VEGFR2), from where various cell types comprising vessels, including endothelial cells and vascular smooth muscles cells are formed [68]. From these findings, it can be speculated that ETV2 may have high potency for generating vascular organoids or vascular graft comprised with various cell types originated from autologous origin. Combined with conventional interventional therapies, ETV2-based therapeutics will provide an innovative and effective therapeutic modality for intractable vessel diseases.

The importance of cost effectiveness should be considered when creating new treatments. Although it is too early to begin clinical applications, it is important to consider that ETV2-based strategy should be affordable. Compared with other novel drug biological products (e.g., advanced therapeutic medicinal products such as genes, cells or combined therapies), mmRNA-based ETV2 delivery based on chemical methods (lipid nanoparticles, liposomes, polymer) [69, 70] would be preferred for rebuilding damaged vessels. Indeed, the efficiency and safety of mmRNA with proper adjuvants have become a novel platform in industry, due to the versatility of swift optimization without causing immunogenicity. This suggests that synthetic/modified ETV2 mRNA can become a safe, profitable and practically affordable strategy for vessel diseases [71, 72].

### RNA-based direct reprogramming

Despite promising outcomes from experimental findings, strategies for the generation and reprogramming of clinically compatible ECs by ETV2 needs further optimization. Transcription factors including ETV2 have been delivered to cells or mice via viral systems, which can cause genomic instability and potential unexpected biological consequences, such as tumorigenesis [73]. Thus, methods avoiding such risks must be utilized to permit the therapeutic function of ETV2 for clinical use. Introducing mRNA into target cells has gained scientific interest for cell fate changes or therapeutic purposes because this biomolecule shows virtually no risk of genome integration, and no further transcription in the cytoplasm is needed [74, 75]. However, the applicability of mRNA has been hampered because of its instability and potential immunogenicity. These limitations are now being overcome by mmRNAs, which have a wide range of modifications in nucleotide, untranslated regions (UTRs), poly-A tail and 5′-capping 7-methylguanine, all of which improve the stability of mRNAs while reducing immunogenicity [76]. Since the first report on the use of mmRNA for reprogramming fibroblast into iPSCs, and their subsequent differentiation into myogenic cells [77], various studies have demonstrated that this approach is non-immunogenic, non-integrating and efficient for cell fate change [74]. In particular, the versatility of mmRNA in direct cell fate conversion has already been demonstrated in several cell types [78]. Corritore et al. demonstrated that human pancreatic duct-derived cells which are epithelial-origin could transdifferentiate into insulin-secreting β-cells through transfection with an mmRNA of V-Maf musculoaponeurotic fibrosarcoma oncogene homolog A, a basic leucine zipper transcription factor that regulates insulin expression in mature β-cells [79]. Additionally, such β-cells ameliorated hyperglycemia upon transplantation into the subcapsular space of diabetic SCID-beige mice. Another study showed that neural precursors can be generated from non-neuronal cells (adult HDFs) through with mmRNAs of SOX2 and PAX6, which are key regulators in neural development [80]. Furthermore, these cells acquired cellular and molecular phenotypes of GABAergic or glutamatergic neurons, depending on the differentiation protocol. These studies would warrant the use of mmRNA of ETV2 in direct cell reprogramming in vitro and in vivo. However, no study so far has reported a success in the direct reprogramming of somatic non-ECs to ECs by mmRNAs, including *ETV2* mmRNA.

### Chemical-mediated reprogramming

Another way of producing desired cell types is using chemicals. Chemical reprogramming is advantageous over conventional protocols using genetic factors in that it can provide better temporal/dosage control and is less immunogenic and tumorigenic [81]. Additionally, the underlying mechanisms for the action of chemicals in animal cells are often well identified, thus enabling one to determine how the reprogrammed target cells can be biochemically obtained [82]. An early investigation showed that mouse fibroblasts were converted into NSCs by the inhibition of GSK3β, MEK and TGF-β signaling in the presence of epigenetic regulators (VPA, Bix01294 and RG108) and a cell death blocker (vitamin C). These NSCs differentiated into neural cells, including astrocytes, oligodendrocytes and neurons in vitro and in vivo [83]. Since this finding, success in converting non-parenchymal cells (e.g., fibroblasts) into various target cells (hepatocytes, cardiomyocytes and skeletal muscles) under a specific set of chemicals has been reported [84]. A recent study also demonstrated that chemical reprogramming can convert cell fate via an intermediate stage. Upon inhibition of GSK3 and ALK5 signaling with simultaneous c-AMP stimulation, fibroblasts underwent epigenetic changes reaching a plastic stage that may possess multilineage potential, after which they further differentiated into diverse cell types including neural or skeletal cells [85]. Similarly, the conversion of human fetal lung fibroblasts and foreskin fibroblasts into cardiomyocytes upon treatment with nine compounds, probably through a mesoderm stage, has been reported [86]. A distinct but partly overlapping chemical cocktail induced neurons from healthy human foreskin fibroblasts and skin fibroblasts from patients with familial Alzheimer’s disease [87], highlighting the clinical potential of chemical reprogramming. Although the generation of chemically reprogrammed parenchymal cells (e.g., cardiomyocytes and neurons) from fibroblasts has been successful [86–88], the efficiency of chemical reprogramming is low [89], and chemical approaches have not been applied to date in endothelial reprogramming. Considering that some small molecules such as ROCK inhibitors, that GSK3 inhibitors and forskolin are the usual suspects for cell reprogramming, and that the generation and maturation of reprogrammed ECs by ETV2 require TGFβ-inhibition or VPA treatment [10, 49], a cocktail of chemicals with such small molecules would be applicable for endothelial reprogramming. Alternatively, combining chemical treatment with *ETV2* activation via either a Crispr/Cas9 system or *ETV2* mmRNAs encapsulated in lipid nanoparticles (LNPs) (see next paragraph) would increase the efficiency of endothelial reprogramming for basic research and clinical studies.

### Gene delivery

Gene delivery systems other than retroviral and lentiviral systems still hold promise and should be optimized to enhance the clinical feasibility of *ETV2*-mediated therapy. Currently, the AAV system is being tested for treating hereditary neuron, muscle, eye and liver genetic disorders [90]. In contrast, attempts to evaluate its clinical use for other major organs including the heart, kidneys and lungs are still lacking [90, 91]. The AAV system was found to be efficient in reprogramming somatic cells into iPSCs in vivo after their administration with reprogramming factors in mice, suggesting that the AAV system may provide efficient tool for in vivo reprogramming [92]. Indeed, the feasibility of AAV-*Etv2* in vascular disease was also demonstrated. In preclinical study, Lee et al. found that the local injection of AAV-*Etv2* into rat MI hearts led to increased vessel growth, reduced scar formation and enhanced cardiac functions [59]. Other parameters including the serotypes of capsid proteins and packaging capacity should be taken into consideration to increase its success in clinical use [90]. Another promising delivery vehicle is Sendai virus (SeV), an RNA virus that does not integrate into the host genome [93]. SeV-mediated gene deliveries have been conducted to treat diverse diseases and have a demonstrated ability to reprogram cell fate [94, 95]. For example, a SeV vector expressing the cardiac reprogramming factors (*Gata4, Mef2c* and *Tbx5*) directly reprogrammed mouse fibroblasts and human cardiac fibroblasts into cardiomyocytes. The injection of these factors through SeV into MI hearts converted cardiac fibroblasts into cardiomyocytes and improved cardiac functions and reduced fibrosis [95]. Therefore, these results support the likelihood that SeV-*ETV2* can serve as an efficient and safe means for direct reprogramming of non-ECs to ECs.

Since the first report of liposome synthesis [96], lipid nanoparticles (LNPs) have been widely tested in many clinical trials to deliver therapeutic substances. As important nanocarriers, LNPs can transport hydrophobic drugs and hydrophilic molecules including small chemicals, nucleic acids and proteins. They have been used for delivering antitumor, anti-inflammatory, antifungal drugs and antibiotics [97]. Various types of LNP-based mRNA vaccines have been developed for clinical trials, including those for viral diseases (rabies, Zika virus, cytomegalovirus, influenza, COVID-19, etc.) and cancers (melanoma, ovarian cancer, glioblastoma, etc.) [76, 97]. Considering mRNA’s proven biosafety and ease of preparation, coupling *ETV2* mmRNA with LNPs for controlled release into host tissues would be an ideal option to overcome current limitations of viral gene delivery systems.

### Engineering vascular organoids/tissues using ETV2

Human organoids have become essential tools for understanding organ development and disease progression, and testing drug efficacy [98]. Despite the progress on establishing various organoids, current protocols are suboptimal partly due to the lack of vasculatures to support the growth and maturation of organoids [99]. Thus, vascularized organoids will provide more physiologically relevant models that mimic in vivo counterparts. A recent study has reported that ECs derived from hESCs overexpressing *ETV2* successfully remodeled to become vascular structures in human cortical organoids (hCOs) [100]. Functionally, vascularized hCOs outperformed avascular hCOs in blood–brain barrier functions, tight junction integrity and the abundance of capillary network vessels capable of perfusion into host circulation in immunodeficient mice. The potential of ETV2 in generating vascular grafts was also demonstrated. Palikuqi et al. have shown that the transient induction of *ETV2* by lentiviral particles resets mature ECs (HUVECs) to an embryonic-like “reset” state, which allows vasculogenesis more efficient. Importantly, these reset ECs formed perfusable and hemodynamic vessels upon being mixed with specific extracellular matrix and were able to establish a vascular network that integrates with tissue-specific parenchymal cells. Functionally, the number of vessels in colon cancer organoids and microfluid-based pancreatic islet organoids was increased [101]. Other than reprogramming strategies, attempts for generating transplantable organs with human endothelium were reported in an animal biotechnology study. Das et al. reported that, upon being complemented with human iPSCs, pig preimplantation embryos deficient in *ETV2* developed human vasculature at E17-18 [102]. This study showed the non-redundant function of ETV2 in vessel formation in a large animal and suggests that chimeric pig organs with human vasculature may become a novel source of various donor biocompatible organs with human endothelium. However, further investigations are needed to enable these chimeric piglets to develop to full term or at least to a stage where their organs can be procured for transplantation studies.

### Challenges and possible strategies for clinical use of ETV2

The non-redundant and essential function of ETV2 in vascular development, regeneration and endothelization of non-ECs has raised the possibility of its effective clinical use [103]. However, there are several roadblocks to its clinical application. As discussed above, the prominent concern is that dormant ECs in adult tissue may not readily express co-activators/repressors or epigenetic activators for proper function of ETV2. Thus, it is crucial to identify the detailed molecular mechanisms by which ETV2 functions. For example, the comprehensive profiling on upstream regulators of ETV2, epigenetic changes on ETV2, ETV2 binding proteins and ETV2-mediated epigenetic regulations would advance our understanding of ETV2 and increase its therapeutic potential.

In clinical procedures, the most favorable route for drug administration is via venous flow. However, this often causes rapid degradation by plasma enzymes or inactivation after interaction with plasma protein [104, 105]. Furthermore, intravenous administration is often a suboptimal route for many cardiovascular disorders [106, 107]; thus, methods that enable targeted delivery are often needed. Accordingly, engineered nanoparticles/LNPs/exosomes/AAV encapsulated with ETV2 and its partners (in the form of mRNA, mmRNA, siRNA) that can target vessel lesions without provoking immune or inflammatory responses should be considered for therapeutic purpose [108]. Another challenge for clinical usage of ETV2 in vessel disease may rise from the complexed microenvironment where ECs are located. The structure and physiology of vessels vary depending on its classification (e.g., arteries vs veins) and the tissue/organ types. Moreover, many vasculopathies present with comorbidities (tumor, diabetes, hypertension, obesity, etc.). Thus, it is difficult to create ETV2-based therapies that target certain vasculature in various contexts. Microneedle-based nanotechnologies for local, controlled release of these potential drug products would be another possible alternative for clinical use to be considered [109, 110]. However, it is also important to note that comprehensive understanding of the safety of the aforementioned gene delivery methods is mandated. Indeed, one preclinical study showed that the transgene loaded in recombinant AAV vector inserted into host genome and was clonally expanded [111], although this has not yet been seen in human. Currently, no information is available regarding therapeutic efficiency of ETV2 in treating vascular disease since studies have only been performed in animal models to determine the role of ETV2 in cardiovascular disease. Thus, more systematic and controlled experiments would be warranted.

## Conclusion

**Fig. 2:**
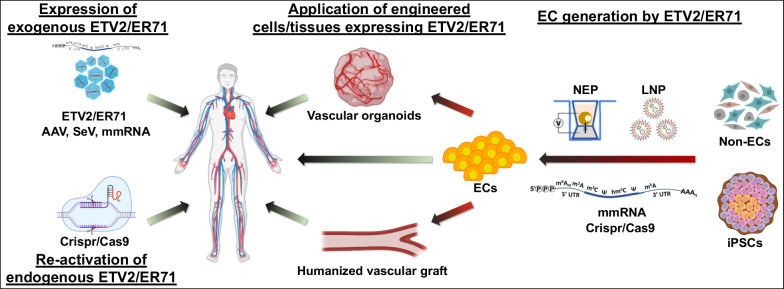
Strategies for vessel regeneration by ETV2-driven reendothelialization. ETV2 may be exogenously administrated into vascular legions in the form of AAV, SeV or mmRNA. Alternatively, endogenous ETV2 can be reactivated via Crispr-Cas9. Therapeutic ECs of autologous origin can be generated from non-ECs or iPSCs by expressing ETV2 mmRNA, non-integrating gene delivery systems (e.g., NEP or LNP) or transactivating ETV2 by Crispr/Cas9. For tissue replacement therapy, these reprogrammed ECs can be either directly administrated or used for generating biocompatible organs/organomimetics such as engineered vascular grafts or vascular organoids. *AAV* Adeno-associated virus; *Crispr-Cas9* Clustered regularly interspaced short palindromic repeats (CRISPR)-CRISPR-associated protein 9; *ECs* Endothelial cells; *ETV2* Ets variant 2/ets-related protein 71; *iPSCs* Induced pluripotent stem cells; *LNP* Lipid nanoparticle; *mmRNA* Modified messenger RNA; *NEP* Nanochannel electroporation; *SeV* Sendi virus. Created in BioRender.com

ETV2 as a pioneer factor for EC development has a potent ability to generate ECs from diverse cell sources. Further investigations on how to design clinically compatible forms of ETV2 and deliver them to targeted cell types or tissues will be required to grasp the therapeutic potential of ETV2 in treating vascular diseases (Fig. 2). Additionally, deciphering the molecular mechanisms of epigenetic changes (histone modifications), DNA methylation and chromatin accessibility along with single-cell omics will significantly advance our understanding of ETV2-mediated cell reprogramming.

## Data Availability

Data sharing is not applicable to this article as no datasets were generated or analyzed during the current study.

## Abbreviations

AAV: Adeno-associated virus
ACs: Amniotic fluid-derived cells
ACTC1: Actin alpha cardiac muscle 1
ALK5: Activin receptor-like kinase 5
ASCL1: Achaete-scute family basic helix–loop–helix transcription factor 1
ARF: ADP-ribosylation factor 1B
BRN2: Bruno-like 2
c-AMP: Cyclic adenosine monophosphate
CAS9: CRISPR-associated protein 9
CD31: Cluster of differentiation 31 (also known as PECAM1)
CD43: Cluster of differentiation 43 (also known as leukosialin or sialophorin)
CD73: Cluster of differentiation 73 (also known as 5′-nucleotidase)
CD133: Cluster of differentiation 133 (also known as prominin 1)
CDH5: Cadherin 5 (also known as VEcadherin)
C/EBPα/β: CCAAT enhancer binding protein alpha/beta
c-KIT: Receptor tyrosine kinase (also known as stem cell factor receptor, CD117)
c-MYC: Cellular myelocytomatosis oncogene
COUP-TFII: Chicken ovalbumin upstream promoter transcription factor 2
COVID: Coronavirus disease
CRISPR: Clustered regularly interspaced short palindromic repeats
CTNT: Cardiac troponin T
E: Embryonic day
EB: Embryoid body
ECs: Endothelial cells
EGFL7: Epidermal growth factor-like domain multiple 7
EpCAM: Epithelial cellular adhesion molecule
ERG: ETS-related gene
ESCs: Mouse embryonic stem cells
ETS: E26-alfalfa mosaic virus oncogene cellular homolog, a transcription factor
ETV2: ETS variant 2 (also known as ER71 or etsrp)
FLI1: Friend leukemia virus integration 1
FLK1: Fetal liver kinase-1
FOXA3: Forkhead box protein A 3
FLT4: Feline McDonough sarcoma related receptor tyrosine kinase 4 (also known as VEGFR-3)
FOXO1: Forkhead box O1
GATA1/2: GATA-binding factor ½
GBM: Glioblastoma multiforme
GSK3: Glycogen synthase kinase
GTP: Guanosine triphosphate
hADSC: Human adipose-derived stem cells
HAND2: Heart and neural crest derivatives expressed 2
hCOs: Human cortical organoids
HDFs: Human dermal fibroblasts
hESCs: Human ESCs
Hnf1α: Hepatocyte nuclear factor-1 alpha
HUVEC: Human umbilical vein endothelial cell
iECs: Induced ECs
iPSCs: Induced pluripotent stem cells
iVECs: Induced vascular ECs
KDR: Kinase insert domain receptor (also known as FLK1 or VEGFR2)
KLF2/4: Kruppel-like factor 2/4
LMO2: Lim domain only 2
LNPs: Lipid nanoparticles
MAFA: Musculoaponeurotic fibrosarcoma basic leucine zipper transcription factor A
MEF: Mouse embryonic fibroblasts
MEF2C: Myocyte enhancer factor 2C
MEK: Mitogen-activated protein kinase (MAPK)/extracellular signal-regulated kinase (ERK) kinase
mESCs: Mouse ESCs
MI: Myocardial infarction
mmRNA: Modified mRNA
MO: Morpholino
MPCs: Mesodermal progenitor cells
MYCT1: MYC target 1
MyoD: Myogenic differentiation 1
MYT1L: Myelin transcription factor 1-like
NGN3: Neurogenin-3
NSCs: Neural stem cells
OCT3/4: Octamer-binding transcription factor 3/4
OVOL2: Ovo-like zinc finger 2
Pax6: Paired box 6
PD1: Programmed death 1
PDGFRα: Platelet-derived growth factor receptor A
PDX1: Pancreatic and duodenal homeobox 1
PROX1: Prospero homeobox 1
RHOJ: Ras homolog family member J
ROCK: Rho-associated protein kinase
SCA1: Stem cell antigen 1
SeV: Sendai virus
siRNA: Small interfering RNA
SOX2: SRY-box transcription factor 2
TAECs: Tumor-associated ECs
TAL1: T-cell acute lymphocytic leukemia protein 1 (also known as SCL)
TBX5: T-box 5
TET2: Ten–eleven translocation 2
TGF: Transforming growth factor
TEK: Tunica interna endothelial cell kinase
TIE2: Tunica interna endothelial cell kinase 2
TRA1: Tumor rejection antigen 1
UTR: Untranslated region
VEGFR2: Vascular endothelial growth factor receptor 2
VPA: Valproic acid
Wnt: Wingless/integrated
